# Estimation of Mycophenolic Acid Exposure in Heart Transplant Recipients by Population Pharmacokinetic and Limited Sampling Strategies

**DOI:** 10.3389/fphar.2021.748609

**Published:** 2021-11-19

**Authors:** Xipei Wang, Yijin Wu, Jinsong Huang, Songgui Shan, Mingjie Mai, Jiade Zhu, Min Yang, Dewei Shang, Zheng Wu, Jinhua Lan, Shilong Zhong, Min Wu

**Affiliations:** ^1^ Research Center of Medical Sciences, Guangdong Provincial People’s Hospital, Guangdong Academy of Medical Sciences, Guangzhou, China; ^2^ Guangdong Provincial Key Laboratory of Clinical Pharmacology, Guangdong Cardiovascular Institute, Guangdong Provincial People’s Hospital, Guangdong Academy of Medical Sciences, Guangzhou, China; ^3^ Department of Cardiac Surgery, Guangdong Cardiovascular Institute, Guangdong Provincial People’s Hospital, Guangdong Academy of Medical Sciences, Guangzhou, China; ^4^ Department of Pharmacy, Guangdong Provincial People’s Hospital, Guangdong Academy of Medical Sciences, Guangzhou, China; ^5^ Department of Pharmacy, The Affiliated Brain Hospital of Guangzhou Medical University, Guangzhou, China; ^6^ School of Biology and Biological Engineering, South China University of Technology, Guangzhou, China; ^7^ Guangdong Provincial Key Laboratory of Coronary Heart Disease Prevention, Guangdong Cardiovascular Institute, Guangzhou, China

**Keywords:** heart transplantation, enzyme multiplied immunoassay technique, maximum a posteriori Bayesian estimation, limited sampling strategy, multilinear regression analysis, population pharmacokinetics, mycophenolic acid

## Abstract

**Purpose:** The aim of this study is i) to establish a strategy to estimate the area under the curve of the dosing interval (AUC_0–12h_) of mycophenolic acid (MPA) in the heart transplant recipients and ii) to find the covariates that significantly affect the pharmacokinetics of MPA exposure.

**Methods:** This single-center, prospective, open-label, observational study was conducted in 91 adult heart transplant recipients orally taking mycophenolate mofetil dispersible tablets. Samples collected intensively and sparsely were analyzed by the enzyme-multiplied immunoassay technique, and all the data were used in PPK modeling. Potential covariates were tested stepwise. The goodness-of-fit plots, the normalized prediction distribution error, and prediction-corrected visual predictive check were used for model evaluation. Optimal sampling times by ED-optimal strategy and multilinear regression (MLR) were analyzed based on the simulated data by the final PPK model. Moreover, using intensive data from 14 patients, the accuracy of AUC_0–12h_ estimation was evaluated by Passing–Bablok regression analysis and Bland–Alman plots for both the PPK model and MLR equation.

**Results:** A two-compartment model with first-order absorption and elimination with a lag time was chosen as the structure model. Co-medication of proton pump inhibitors (PPIs), estimated glomerular filtration rate (eGFR), and albumin (ALB) were found to significantly affect bioavailability (F), clearance of central compartment (CL/F), and the distribution volume of the central compartment (V_2_/F), respectively. Co-medication of PPIs decreased F by 27.6%. When eGFR decreased by 30 ml/min/1.73 m^2^, CL/F decreased by 23.7%. However, the impact of ALB on V_2_/F was limited to MPA exposure. The final model showed an adequate fitness of the data. The optimal sampling design was pre-dose and 1 and 4 h post-dose for pharmacokinetic estimation. The best-fit linear equation was finally established as follows: AUC_0–12h_ = 3.539 × C_0_ + 0.288 × C_0.5_ + 1.349 × C_1_ + 6.773 × C_4.5_.

**Conclusion**: A PPK model was established with three covariates in heart transplant patients. Co-medication of PPIs and eGFR had a remarkable impact on AUC_0–12h_ of MPA. A linear equation was also concluded with four time points as an alternative way to estimate AUC_0–12h_ for MPA.

## Introduction

Mycophenolic acid (MPA) is the active metabolite of the prodrug mycophenolate mofetil (MMF), with a high oral bioavailability (95%) (Bullingham et al., 1996). MPA selectively inhibits inosine monophosphate dehydrogenase in *de novo* purine synthesis in T and B lymphocytes. As an antimetabolite drug, MPA is one of the most commonly prescribed drugs in immunosuppression therapy to prevent graft rejection after kidney, lung, liver, and heart transplantation (Bergan et al., 2021). It has a protein binding rate of about 97% and is mainly metabolized by uridine diphosphate glucuronosyltransferase (UGT) into the inactive 7-*O*-glucuronide (MPAG) metabolite (Bullingham et al., 1998). It exhibits enterohepatic circulation (EHC) during which MPAG returns to the small intestine and is degraded into MPA by microorganisms, and MPA is re-absorbed into the circulatory system. MPA is eliminated through the kidneys mainly as MPAG (Bergan et al., 2021). Adequate MPA exposure could effectively prevent graft rejection, while oversuppression may increase the cancer risk in organ transplant recipients (Huo et al., 2020). The area under the curve of the dosing interval (AUC_0–12h_) of MPA is considered a reliable biomarker for graft rejection (Tett et al., 2011). It was reported that AUC_0–12h_ values below 36 mg h /L were related to heart transplantation rejection (Dösch et al., 2006; Figurski et al., 2012). However, the method to directly calculate AUC_0–12h_ needs intensive sample collection, which is difficult to implement in clinical application.

Previously, the methods used to estimate the MPA AUC_0–12h_ in heart transplant recipients were investigated, including limited sampling strategies with multiple linear regression (MLR), population pharmacokinetic (PPK), and machine learning (ML) approaches (Bergan et al., 2021; Woillard et al., 2021). MLR was used in the estimation of MPA AUC_0–12h_ in several published studies in heart transplant recipients (Ting et al., 2008; Baraldo et al., 2009; Pawinski et al., 2009). The linear equations were convenient to use but with different sampling time points and large variability between studies. Most importantly, there were systematic errors between the bioanalysis methods of MPA concentrations. The measurements by the enzyme-multiplied immunoassay technique (EMIT) were higher than those by high-performance liquid chromatography (HPLC), with a positive bias over 24% (Kunicki et al., 2015; Bergan et al., 2021). Based on the HPLC-measured MPA concentrations from Chinese adult heart transplant recipients, a four timepoint linear model (0.5, 2, 4, and 6 h post-dose) was generated (Xiang et al., 2021). However, it could not be applied to the MPA concentrations measured by the EMIT for the systematic errors, which was not negligible. Moreover, fixed sampling time points in the linear model made it difficult to deal with clinically variable situations, such as missing data and collection time deviation.

PPK modeling is more flexible for sample collection and has been widely used in the estimation of MPA AUC_0–12h_ in lung, liver, and kidney transplantations (Kiang and Ensom, 2018). However, PPK studies in heart transplant recipients are limited. A website platform (ISBA 3.0) provides MPA AUC_0–12h_ estimation in Caucasian patients by a Bayesian approach based on three samples (https://abis.chu-limoges.fr/). A previously published study based on the PPK approach using both parametric and nonparametric methods resulted in an equally accurate estimation of AUC_0–12h_ (Woillard et al., 2015). However, no covariate was previously found in the heart transplant recipients. The main factors that influence the pharmacokinetics of MPA were important for personalized therapy. ML models built based on a large pooled dataset from organ transplant recipients and patients with autoimmune disease treated with various immunosuppressant co-medications [such as cyclosporine and tacrolimus (TAC)] yielded better performances in AUC_0–12h_ estimation than the Bayesian approach. However, it was not built on a biological basis, and it was even less flexible than the PPK approach with respect to the number of samples and sampling time points (Woillard et al., 2021). Both linear regression and PPK modeling have their advantages in clinical application. Linear equations are easy to use and popularize. PPK features fewer sampling limitations, and its application in clinical practice is flexible. However, no study provided a systematic strategy to adapt to clinical variability using both linear regression and a PPK approach.

The aim of the present study was i) to establish a systematic strategy for MPA AUC_0–12h_ estimation and ii) to investigate the impact of the main factors on the MPA AUC_0–12h_ in Chinese adult heart transplant recipients. This gives a reference to the doctors for the selection of a convenient and accurate method for MPA AUC_0–12h_ estimation, taking into account key factors when making dose adjustments.

## Materials and Methods

### Study Design and Patients

**FIGURE 1:**
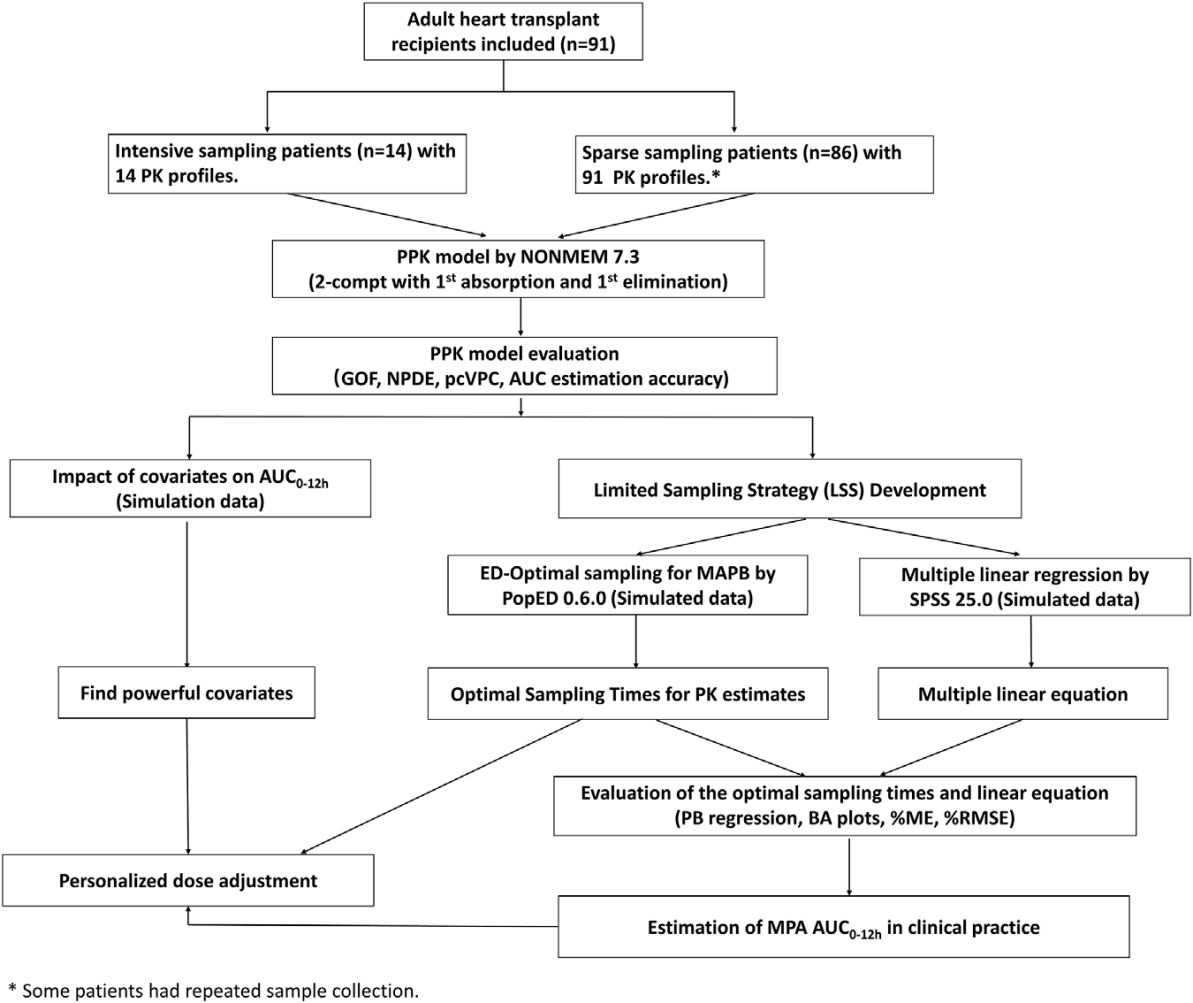
Flowchart of the study. PPK, population pharmacokinetic; AUC_0–12h_, the area under the curve of the dosing interval; MPA, mycophenolic acid; GOF, goodness-of-fit; NPDE, normalized prediction distribution error; pcVPC, prediction-corrected visual predictive check; MAPB, maximum a posteriori Bayesian; PB regression, Passing–Bablok regression; BA plot, Bland-Altman plot; %ME, mean error in percentage; and %RMSE, percent root mean squared error.

This was a single-center, prospective, open-label, observational study. The entire study design is shown in Figure 1. Patients were aged ≥18 years and underwent their first heart transplantation surgery at Guangdong Provincial People’s Hospital and received triple maintenance immunosuppressive therapy comprising MMF, TAC, and corticosteroids. Patients undergoing combined organ transplantation were excluded. The study protocols were approved by the Research Ethics Committee of Guangdong Provincial People’s Hospital [2018478H (R1)], and all participants provided written informed consent before inclusion. The study protocol was registered in the Chinese Clinical Trial Registry (No. ChiCTR2000030903). Volunteers were the sole source of transplants in the current study. There were no executed donors in the current study. Generally, in China, doctors select heart donors under the age of 50. The mean age of the donors in the present study was 35.1 ± 9.4 years, and six donors were females. The donor cause of death was trauma, cerebrovascular accident, brain tumor, or hypoxic brain injury.

MMF dispersible tablets (Cycopin®, manufactured by Huadong Medicine Co., Ltd.) were orally administered at a maintenance dose of 250–750 mg twice daily (bid). TAC (Prograf®) was dosed at 0.5–4 mg once or twice a day after transplantation and therapeutic drug monitoring was conducted to achieve the target concentration. Methylprednisolone (20 mg) was administered during the operation and was switched to 8 mg in the following days. Sample collection for the measurement of MPA concentrations started at least 7 days post-operation. Full pharmacokinetic profiles were obtained by intensive sampling, drawing 10 blood samples: pre-dose, 0.5, 1, 1.5, 2, 3, 4, 6, 8, and 12 h after the morning dose. For sparse sample collection, blood samples were drawn at 0.5, 1.5, 4, and 9 h post-dose after the morning dose. More than one pharmacokinetic sampling cycle might be collected in one patient.

Patient information was collected during sample collection from the medical electronic records, including demographic information, biochemical indices, concomitant medications, TAC concentrations and daily dose, and postoperative time (POT). Proton pump inhibitors (PPIs) included omeprazole injections or gastro-resistant tablets and pantoprazole injections or enteric-coated tablets.

### Sample Collection and Bioanalytical Assay

Blood samples were collected in EDTA-coated tubes and centrifuged at room temperature (2,000 × *g* for 10 min) to separate plasma, which was analyzed within 24 h after collection. MPA concentrations were detected by the EMIT using MPA kits on the Viva-E system (Siemens Healthcare Diagnostics, Newark, Del, United States) according to the manufacturer’s guidelines. The linear range of MPA concentrations was 0.1–15 mg/L. The lower limit of quantification (LLOQ) was 0.1 mg/L. At the control levels of 1, 7.5, and 12 mg/L, the precisions were 5–6%, 3.1–4.5%, and 4.3–6.5%, respectively.

### Pharmacokinetic Modeling

The intensive and sparse concentration data were pooled and simultaneously fitted using a nonlinear mixed-effects modeling software program (NONMEM, version 7.3.0, ICON Development Solutions, MD, United States). The units of MMF doses and MPA concentrations were unified as moles by multiplying the molecular weights. The first-order conditional estimation with interaction (FOCEI) method was used throughout the entire model-building process. One- or two-compartment models with time-lagged first-order absorption and first-order elimination or a two-compartment model with EHC were tried as the structure model. Oral bioavailability (F) was fixed as 0.95 as in Bullingham et al. (1996) and Armstrong et al. (2005). Inter-individual variability (IIV) of each PK parameter was modeled using the following exponential error model:

$$P_{i}=P_{tv}\cdot e^{\eta_{i}},$$ (1)

where *P*_i_ represents the PK parameter of the ith individual, *P*_tv_ represents the typical population value, and *η*_i_ is the inter-individual random effect with mean 0 and variance ω^2^. The covariance between IIV values was estimated using a variance–covariance matrix.

The influence of covariates, including sex, age, body weight (WT), height (HT), body mass index (BMI), hemoglobin (HGB), hematocrit (HCT), serum creatinine (Scr), serum albumin (ALB), total protein (TP), blood urea nitrogen (BUN), uric acid (URIC), total bilirubin (TBIL), direct bilirubin (DBIL), diuretic co-medication (1 for yes/0 for no), PPI co-medication (1 for yes/0 for no), and TAC daily dose and trough concentrations on the main pharmacokinetic parameters was evaluated. The estimated glomerular filtration rate (eGFR) was calculated by the Modification of Diet in Renal Disease (MDRD) equation (modified for Chinese): eGFR = 175 × (Scr)^−1.234^ × (Age)^−0.179^ × (0.79 if female) (Ma et al., 2006). Discrete covariates (such as sex and co-medication of PPIs) were modeled as follows:

$$P_{i}=P_{tv}\cdot \theta^{COV_{i}}\cdot e^{\eta_{i}}.$$ (2)

Here, *COV*_i_ represents the discrete covariate value of the ith individual as a binary variable (1 or 0) and Θ represents the factor of the covariate.

For continuous covariates (such as WT and ALB), an exponential or linear model with average covariate values and an adjusting factor were tried as follows:

$$\;\;P_{i}=P_{tv}\cdot {\left(\frac{COV_{i}}{COV_{ave}}\right)}^{\theta }\cdot e^{\eta_{i}},$$ (3)

$$P_{i}=P_{tv}\cdot \left(1+\left(COV_{i}-COV_{ave}\right)\cdot \theta \right)\cdot e^{\eta_{i}}.$$ (4)

Here, *COV*_i_ represents the continuous covariate value of the ith individual, *COV*_ave_ represents the average values of the covariates, and *Θ* represents the factor of the covariate.

The objective function value (OFV) and goodness-of-fit (GOF) plots were used as the criteria for model selection. For forward inclusion, it was considered significant if the OFV decreased more than 6.63 (chi-squared distribution, *d* = 1, *p* < 0.01). Backward exclusion had a stricter criterion of increase in the OFV, that is, OFV >10.83 (chi-squared distribution, *d* = 1, *p* < 0.001).

### PPK Model Evaluation

The adequacy of the final model was assessed using GOF plots, the normalized prediction distribution error (NPDE) and prediction-corrected visual predictive check (pcVPC). 1,000 bootstraps for the 95% confidence intervals (CIs) of the parameters (*n* = 1,000 times) were conducted in Perl-speaks-NONMEM (PsN) version 4.9.0 (psn.sourceforge.net, Uppsala University, Sweden) (Lindbom and Jonsson, 2004). The GOF plots were generated using R software (version 3.6.3) (R Core Team, 2019). The NPDE analysis and plotting were implemented using the NPDE add-on package (version 2.0) (Scrucca et al., 2016). PcVPC was performed by simulations (*n* = 1,000) based on the final PPK model using PsN and plotted by R.

The predictive performance of AUC_0–12h_ by the final PPK model was evaluated using intensive data from 14 patients. The AUC_0–12h_ value of MPA based on the intensive concentrations versus the time profile (marked as AUC_obs_) was calculated directly by the linear trapezoidal rule using the R add-on package PKNCA (version 0.9.2) (Denney et al., 2015). The individual-predicted AUC_0–12h_ (AUC_ipred_) was calculated by the simulated pharmacokinetic profiles with a virtual sampling increment of 0.5 h, using the individual PK parameters estimated by the final PPK model. The bias was expressed as the mean error in percentage (%ME), and precision was evaluated using percent root mean squared error (%RMSE) as in the following equations.

$$\%ME=\frac{1}{N}\sum_{i=1}^{i=N}\frac{AUC_{pred,i}-AUC_{ref,i}}{AUC_{ref,i}}\cdot 100,$$ (5)

$$\%RMSE=\sqrt{\frac{1}{N}\sum_{i=1}^{i=N}{\left(\frac{AUC_{pred,\;i}-AUC_{ref,i}}{AUC_{ref,i}}\right)}^{2}}\cdot 100.$$ (6)

Here, *AUC*_*ref,*__*i*_ is the AUC_obs_ of the *i* ^th^ patient and *AUC*_*pred,*__*i*_ is the AUC_ipred_ of the *i* ^th^ patient. Passing–Bablok regression analysis and Bland–Alman plots were used to estimate the relationship between predicted and observed AUC_0–12h_ values using an R add-on package (MethComp 1.30.0) (Carstensen et al., 2020).

### Impact of Covariates on MPA Exposure

Simulations were performed on the final PPK model to investigate the impact of the significant covariates on MPA exposure. Full pharmacokinetic profiles at steady-state, with time point increments of 0.5 h during a 12-h dosing interval, were simulated at a virtual dose of 500 mg MMF in 24 scenarios, including eGFR values of 30, 60, 90, and 130 ml/min/1.73 m^2^, ALB values of 30, 40, and 60 g/L, and with or without PPIs. The eGFR values were used to simulate patients with severe or mild renal impairment, normal renal function, and augmented renal clearance (ARC). The selected ALB value of 30 g/L was used to simulate patients with hypoproteinemia. The AUC_0–12h_ values were calculated by the linear trapezoidal rule based on population-predicted (PRED) values using R.

### Limited Sampling Strategy Development

For the limited sampling strategy (LLS), we used two methods: 1) optimal sampling times for Bayesian estimation by ED-optimal sampling and 2) a fixed time-point linear equation using MLR.

For the accurate individual pharmacokinetic estimates, an ED-optimal sampling strategy was applied to optimize the sampling time points using the R package PopED (version 0.6.0) (Nyberg et al., 2012). For the current study, the sampling timepoints were constrained to four within 6 h after drug intake, including pre-dose (0 h). To evaluate the accuracy of AUC_0–12h_ estimation based on the optimal sampling times, we performed simulations to generate 100 virtual patients receiving MMF at an oral dose of 500 mg. The covariate values (i.e., eGFR, ALB, and co-medication of PPIs) were generated using the mean and standard deviation of the original patients. Intensive concentrations of the virtual patients during a dosing interval were generated (as a reference dataset) based on the final PPK model. For the optimal sampling dataset, we assumed that the plasma concentration samples were only collected at the optimal sampling times from the reference dataset. AUC_0–12h_ was calculated with the linear trapezoidal rule using R. AUC_ref_ was calculated based on the reference dataset. AUC_opt_ was calculated based on the simulated concentrations by the individual PK parameters estimated by maximum a posteriori Bayesian (MAPB) estimation. We also selected the observed concentrations at the optimal sampling timepointss from the intensive dataset (*n* = 14) and estimated the individual PK parameters by MAPB. The AUC_0–12h_ value was marked as AUC_opt14_. Predictive performance was evaluated by comparing the individual reference values with those estimated based on the optimal sampling dataset, respectively, and the %ME and %RMSE calculated by Eqs 5, 6. Here, *AUC*_*ref,*__*i*_ is AUC_ref_ of the *i*th virtual patient or the AUC_obs_ of the *i*th intensive sampling patient and *AUC*_*pred,*__*i*_ is AUC_opt_ of the *i*th virtual patient or the AUC_opt14_ of the *i*th intensive sampling patient. Passing–Bablok regression analysis and Bland–Alman plots were used to estimate the correlation using R.

For clinical convenience, a fixed time-point linear equation was generated using simulated data based on the original dataset. Using the actual doses and the patients’ pharmacokinetic parameters obtained from the final PPK model, we simulated the complete pharmacokinetic profiles with a time increment of 0.5 h during the dosing interval. The simulated AUC_0–12h_ values were calculated by the linear trapezoidal rule as the reference (marked as AUC_ipred_). With simulated data of each patient, we searched for predictive models of the AUC_ipred_ using multiple stepwise linear regression analysis with a limited sampling window ≤6 h post-dose and a maximum number of four samples. Data were analyzed using the software package SPSS 25.0 for Windows (SPSS Inc., Chicago, IL, United States). We considered linear equations with high coefficients (*r* ^2^) and clinical convenience as acceptable. The variance inflation factor (VIF) was calculated to check for collinearity of the variables, and VIF <10 was preferred. AUC_0–12h_ calculated by the final linear model was marked as AUC_MLR_. The predictive accuracy was evaluated by comparing the AUC_ipred_ to AUC_MLR_ and the AUC_obs_ to AUC_MLR_, respectively. The %ME and %RMSE were calculated by Eqs 5, 6. In the equations, *AUC*_*ref,*__*i*_ is AUC_ipred_ of the *i*th patient and *AUC*_*pred,*__*i*_ is AUC_MLR_ of the *i*th patient. To compare with the observed AUC_0–12h_, *AUC*_*ref,*__*i*_ is found to be AUC_obs_ of the *i*th patient from 14 intensive sampling patients and *AUC*_*pred,*__*i*_ is AUC_MLR_ of the *i*th patient. Passing–Bablok regression analysis was performed, and Bland–Alman plots were generated to evaluate the accuracy of the linear equation.

In addition, optimal sampling based on the time-points in the final MLR equation was also evaluated, for there might be missing samples in clinical practice. If it happened, AUC_0–12h_ could also be estimated by MAPB estimation based on the linear sampling times.

## Results

### Patients’ Characteristics

In total, 508 plasma samples with 507 above LLOQ were obtained from 91 adult heart transplant recipients with 105 pharmacokinetic sampling cycles. Seventy-seven patients had one PK profile, 13 patients had two, and one patient had three PK profiles. The sampling time after operation (POT) was extensive, from 7 days to nearly 3 years. The intensive sampling was collected from 14 patients, and the other data were sparse. The patient characteristics are shown in Table 1. The recipients (seven females) had a median age of 50 (range, 21–74) years and a median bodyweight of 60.0 kg (range, 33.4–95.0 kg). Furthermore, the doses of MMF were in a range of 250–750 mg, co-medicated with TAC dosed from 1 to 8 mg per day.

**TABLE 1:** Demographic characteristics of adult heart transplant recipients for population pharmacokinetic analysis of mycophenolic acid (MPA).

	Median (range) or n (%)
Demographic data	
No. of patients	91 (84 males)
Age (years)	50 (21–74)
Weight (kg)	60.0 (33.4–95.0)
Height (m)	1.65 (1.51–1.78)
body mass index (BMI, kg/m^2^)	21.5 (14.1–50.0)
Drug therapy	
Dose of mycophenolate mofetil	500 mg (250–750 mg) bid
Co-medication of tacrolimus dose (mg/day)	3.0 (1.0–8.0)
Tacrolimus trough concentration (ng/ml)	7.5 (2.2–30.0)
Co-medication of methylprednisolone dose (mg/day)	8 (8–20)
Co-medication of diuretics (n (%))	46 (50.5%)
Co-medication of proton pump inhibitors (PPIs, n (%))	45 (49.5%)
Postoperative time (POT, day)	37 (7–1,067)
Biochemical indices	
Serum creatinine concentration (Scr,mg/dL)	1.21 (0.35–6.55)
Estimated glomerular filtration rate (eGFR, mL/min/1.73 m^2^)	57.2 (6.3–197.1)
Alanine aminotransferase (ALT, U/L)	21.0 (4.0–136)
Blood urea nitrogen (BUN, mmol/L)	8.9 (4.6–60.5)
Total protein (TP, g/L)	66.4 (33.4–85.2)
Albumin (ALB, g/L)	40.50 (28.56–57.90)
Uric acid (URIC, μmol/L)	368.0 (115.1–826.0)
Hematocrit (HCT, L/L)	0.355 (0.207–0.570)
Hemoglobin (HGB, g/L)	117 (71–191)
Percentage of lymphocytes (LYMPH%, %)	17.9 (0.5–49.3)
Total bilirubin, (TBIL, μmol/L)	14.1 (5.8–82.3)
Direct bilirubin, (DBIL, μmol/L)	3.6 (1.2–45.2)

### Final PPK Model

MPA plasma concentrations were adequately fitted using the two-compartment model with lag time (T_Lag_) and three covariates in the final model. The combined residual error model resulted in a proportional error of 26.1% and an additive residual error of 0.144 mg/L. The PPK parameter estimates with 95% CIs are shown in Table 2. The impacts of co-medication of PPIs on the bioavailability (F) of eGFR on the clearance of the central compartment (CL/F) and ALB on the volume of the central compartment (V_2_/F) were significant covariates incorporated in the final PPK model. On the use of PPIs, the F of MPA decreased to 72.4%. The typical value of CL/F was 7.36 L/h. The factor of the eGFR covariate model (*θ* in Eq. 4) was 0.00791. The inclusion of eGFR explained 11.6% of the IIV of CL/F. With an incremental decrease of 30 ml/min/1.73 m^2^ of eGFR, CL/F decreased by 23.7%. The impact factor of ALB on V_2_/F was −7.31 using the exponential covariate model (Eq. 3), which resulted in a 30.8% reduction of IIV of V_2_/F. The Q/F was estimated as 17.0 L/h, and the V_3_/F was as large as 560 L with very high IIV (189.5%). The model-building process is shown in Supplementary Table S1.

**TABLE 2:** Population pharmacokinetic parameters of MPA in the heart transplant recipients (*n* = 91).

PK parameters	Values	RSE (%)	Shrinkage (%)	Results by bootstraps (*n* = 1,000)	
				Median	90% CI
Ka, 1/h	0.781	13.8	—	0.780	0.641–0.964
F	0.95 FIX	—	—	—	—
CL/F, L/h	7.36	6.9	—	7.30	6.39–8.25
V_2_/F, L	5.69	43.4	—	5.78	3.41–12.6
Q/F, L/h	17.0	11.1	—	16.7	13.7–20.7
V_3_/F, L	560	47.0	—	578	320–1,179
T_lag_, h	0.408	7.4	—	0.405	0.297–0.447
*θ*_PPI-F_	0.724	9.9	—	0.720	0.587–0.893
*θ*_eGFR-CL_	0.00791	12.4	—	0.00794	0.00513–0.0112
*θ*_ALB-V2_	−7.31	26.8	—	−7.13	−10.80– −4.54
IIV of Ka, %	41.5	32.6	46.7	41.3	28.7–55.5
IIV of CL/F, %	41.2	15.1	21.0	40.4	22.2–55.5
IIV of V_2_/F, %	186.5	15.1	28.2	180.9	136.3–224.1
IIV of Q/F, %	33.3	50.5	54.9	32.1	5.2–47.0
IIV of V_3_/F, %	189.5	24.1	59.7	189.7	96.8–260.1
IIV of T_lag_, %	13.1	125	65.8	13.7	3.0–24.6
IIV of F, %	22.1	48.9	49.1	21.8	4.1–35.1
Prop. res. error, %	26.1	8.3	19.3	26.0	18.3–33.1
Add. res. error, mg/L	0.144	27.8	23.1	0.141	0.0266–0.338

Ka, absorption constant; CI, confidence interval; CL, clearance of the central compartment; F, bioavailability; V_2_, distribution volume of the central compartment; Q, clearance of the peripheral compartment; V_3_, distribution volume of the peripheral compartment; T_lag_, lag time of absorption; F, bioavailability; IIV, inter-individual variability; Prop. res. error, proportional residual error; Add. res. error, additive residual error; RSE, relative standard error. *θ*_PPI-Ka_: the factor of co-medication of PPI on F as $F=TV_{F}\times \theta_{PPI-F}$ , where PPI equals one if co-medication of PPI during PK sampling, PPI equals 0 if not. *θ*_eGFR-CL_: the factor of eGFR on CL as the exponential model $CL=TV_{CL}\times \left(1+\left(eGFR-57\right)\cdot \theta_{\text{eGFR}-\text{CL}}\right)$ . *θ*_ALB-V2_: the factor of ALB on V_2_ as the exponential model $V_{2}=TV_{V2}\times {\left(\frac{ALB}{40}\right)}^{\theta_{ALB-V2}}$ .

### PPK Model Evaluation

**FIGURE 2:**
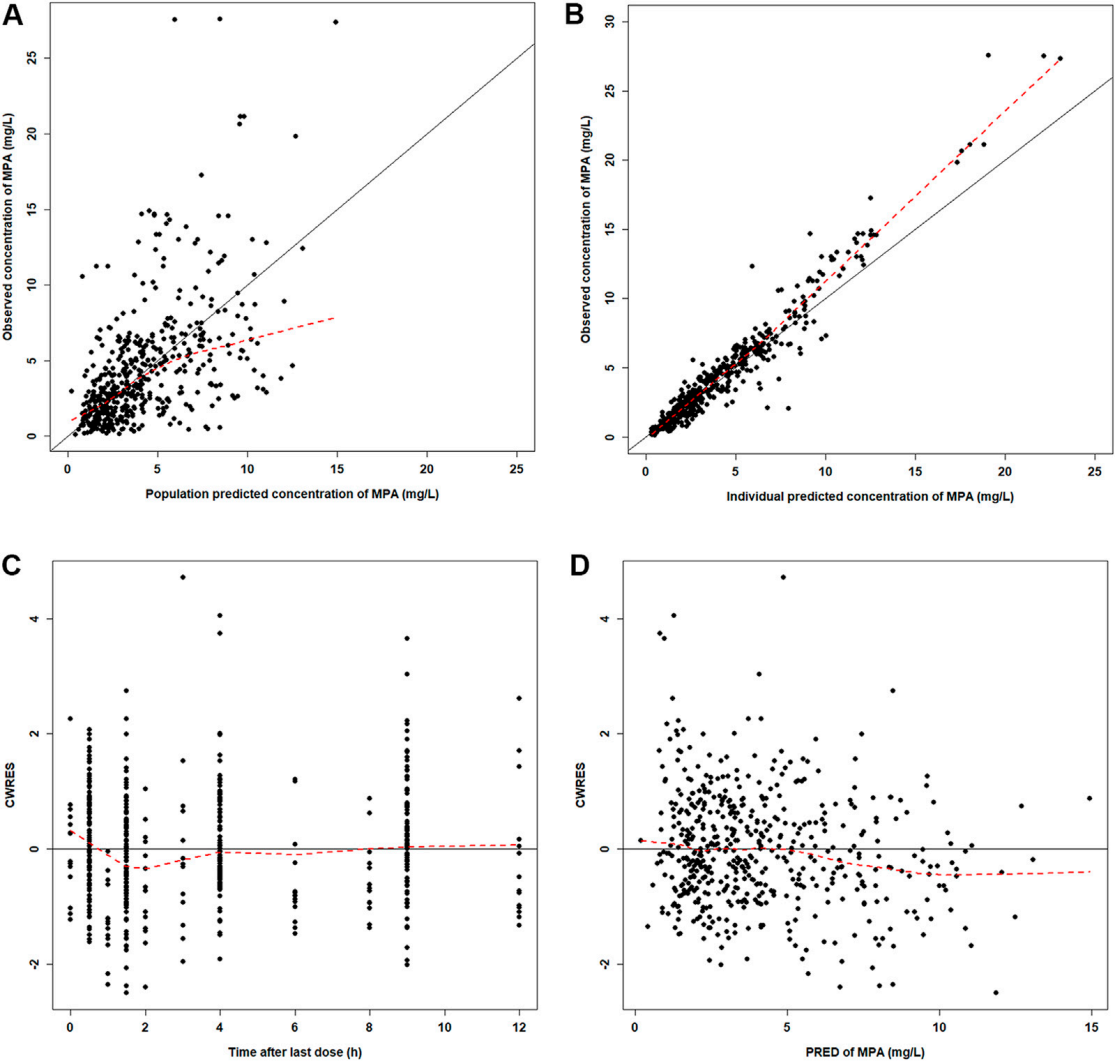
Diagnostic plots for the final PK model. **(A)** Observed versus population-predicted concentrations (DV vs. PRED). **(B)** Observed versus individual-predicted concentrations (DV vs. IPRED). **(C)** Conditional weight residual error versus time (CWRES vs. TIME). **(D)** Conditional weight residual error versus population-predicted concentration (PRED) (CWRES vs. PRED).

**FIGURE 3:**
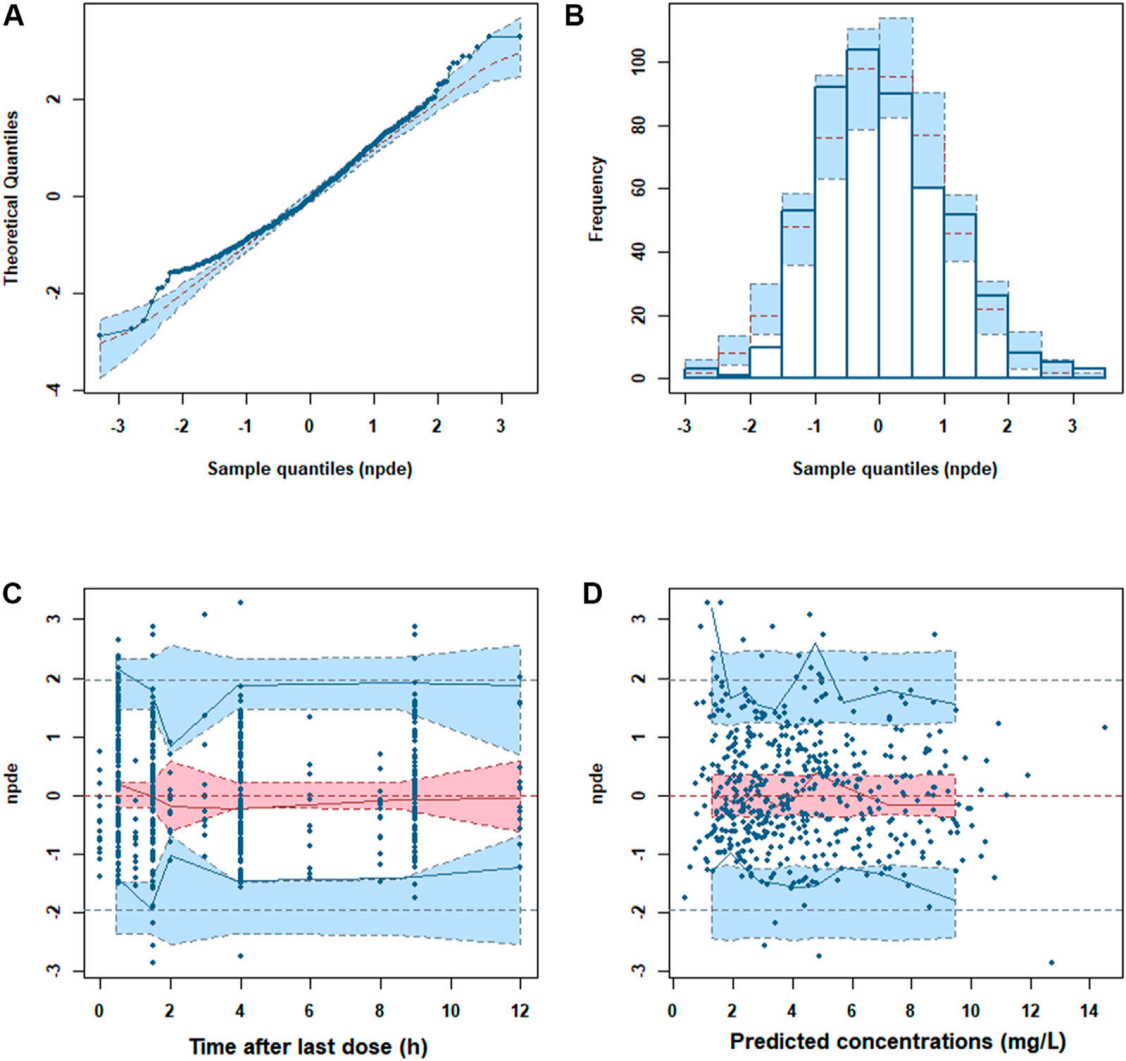
Normalized prediction distribution error (NPDE) metrics of the final model. **(A)** QQ plot of the NPDE. **(B)** Distribution of the NPDE. **(C)** NPDE versus time. **(D)** NPDE versus predicted concentrations.

The GOF plots showed an adequate fitness of the final model (Figure 2). There was a good agreement between population-predicted concentrations (PREDs) and the observed concentrations, except for underestimating the higher observations (Figure 2A). It improved in the individual-predicted concentrations (IPREDs) vs. the observed concentrations. However, a similar underestimation also occurred in the higher concentration range (>15 mg/L). With an acceptable range of the conditional weighted residuals (CWRES), CWRES indicated no obvious trends throughout the time and the predicted concentrations (Figures 2C,D). The NPDE plots are shown in Figure 3. The quantile–quantile (QQ) plot and the distribution histogram of the NPDE showed a mean of 0.0613 and a variance of 0.9812 (*p* = 0.164) (Figures 3A,B). There was no trend for the NPDE versus time (Figure 3C) or the NPDE versus the predicted concentrations (Figure 3D). These results indicated that the final PPK model of MPA was accurate and reliable. The pcVPC plot is shown in Supplementary Figure S1. The pcVPC plot shows that most of the observed concentrations were between the 5th and 95th percentiles of model predictions, except that the predictions were higher than the observations around the peak timepoints (1–2 h post-dose). Generally, the model adequately characterized MPA concentrations.

Compared with the observed AUC_0–12h_ values (AUC_obs_) of the intensive data, the accuracy (%ME) and precision (%RMSE) of the AUC_0–12h_ estimation were 5.0 and 11.8%, respectively. High correlations between the AUC_obs_ and AUC_ipred_ (*r* = 0.977) and most of the differences within 1.96 SD limits are shown in Supplementary Figure S2. It demonstrated that the final PPK model predicted the AUC_0–12h_ values well.

### Impacts of Covariates on MPA Exposure

The simulated AUC_0–12h_ values at the steady-state calculated by the PRED are listed in Table 3. The change of ALB values resulted in few changes in AUC_0–12h_. However, PPI co-medication and eGFR change remarkably affected MPA exposure. With PPIs, AUC_0–12h_ was reduced by approximately 26%, which was uniform with the influence on F. The simulated AUC_0–12h_ values under severe renal impairment, mild renal impairment, and ARC were about 1.60-, 1.23-, and 0.78-fold that for normal renal function, respectively. Without PPIs, severe renal-impaired patients acquired the highest MPA exposure.

**TABLE 3:** Simulated steady-state area under curve (AUC_0–12h_) of mycophenolic acid (MPA) at a dose of 500 mg mycophenolate mofetil bid at different covariate values based on the final PPK model.

PPI co-medication	eGFR (mL/min/1.73 m^2^) ALB (g/L)	30	60	90	130
No	30	82.1	63.0	51.2	40.9
No	40	82.2	63.2	51.3	41.1
No	60	84.8	65.5	53.5	43.0
Yes	30	59.4	45.6	37.0	29.6
Yes	40	59.5	45.7	37.2	29.7
Yes	60	61.4	47.4	38.7	31.1

ALB, albumin; PPI, proton pump inhibitor; eGFR, estimated glomerular filtration rate. The unit of AUC_0–12h_ was mg·h /L.

### Optimal Sampling Times and Linear Model by LSS

**FIGURE 4:**
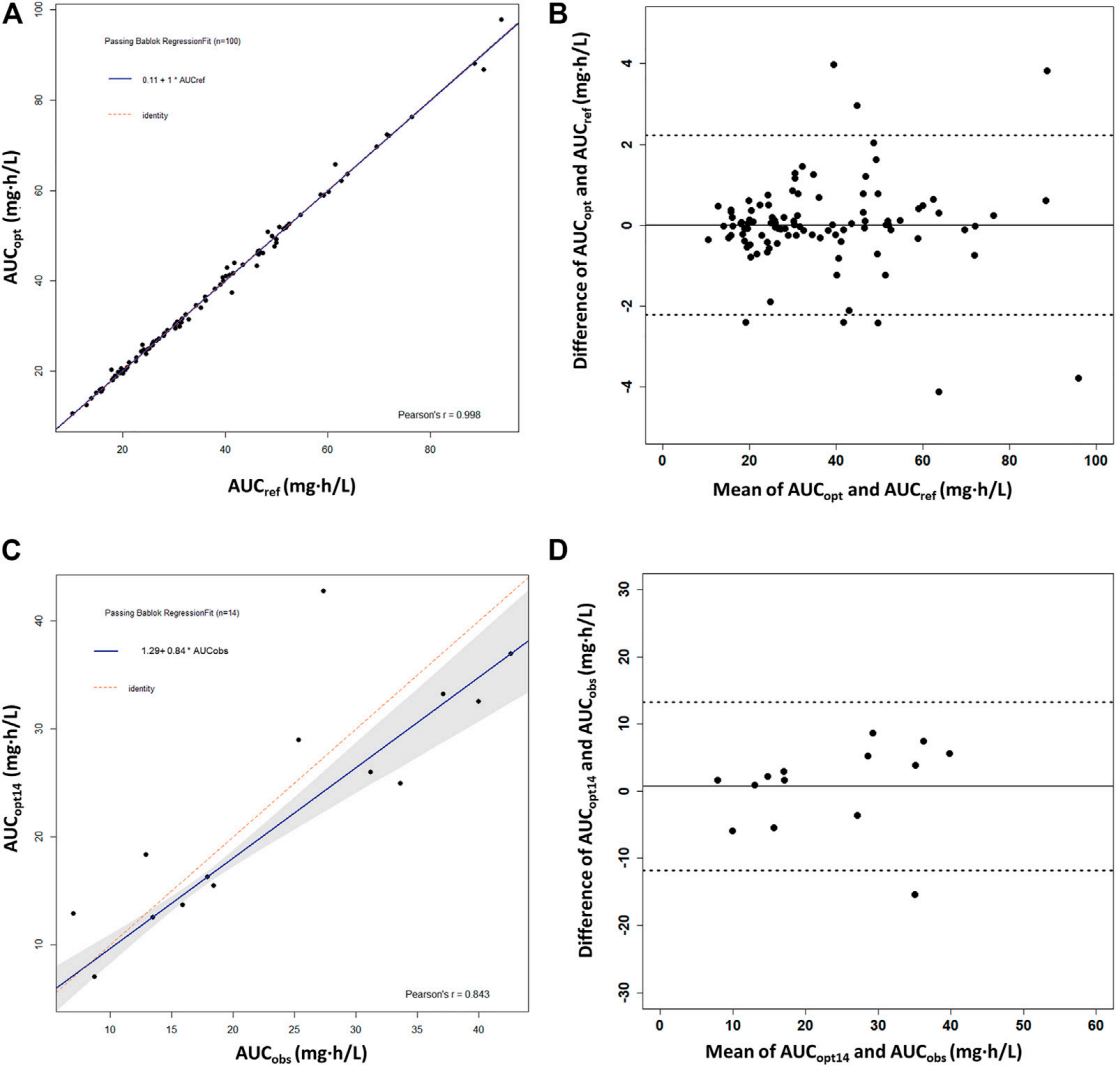
Passing–Bablok regression analysis and the Bland–Altman plot between the reference AUC_0–12h_ and the predicted AUC_0–12h_ of MPA obtained using the optimal sampling times by maximum a posteriori Bayesian (MAPB) estimation in heart transplant recipients. **(A,B)** Comparison between AUC_0–12h_ based on the full PK profiles of the virtual patients (AUC_ref_) and AUC_0–12h_ based on the optimal sampling times (AUC_opt_) (*n* = 100). **(C,D)** Comparison between the observed AUC_0–12h_ from the patients with intensive data as the reference AUC_0–12h_ (AUC_obs_) and AUC_0–12h_ based on the optimal sampling times (AUC_opt14_) (*n* = 14). AUC_0–12h_, the area under the curve of the dosing interval; MPA, mycophenolic acid.

The ED-optimal sampling timepoints included three timepoints: pre-dose and 1 and 4 h post-dose. We found that, except for 1 or 4 h post-dose alone, taking any of the optimal sampling times could obtain accurate AUC_0–12h_ by MPAB estimation, with the value of correlation coefficients above 0.97 (Supplementary Table S2). We got the highest *r* ^2^ (0.9981) with the %ME value of −0.1% and the %RMSE value of 2.9% taking all three sampling timepoints. It showed a high linear correlation between AUC_ref_ and AUC_opt_ (*r* = 0.998) (Figure 4A). The Bland–Altman plot showed that only eight AUC_opt_ values were outside the 1.96SD limit (Figure 4B). The linear coefficient between AUC_obs_ and AUC_opt14_ was 0.843 (Figure 4C), with an acceptable bias (%ME = –3.3%), but with a larger precision (%RMSE = 32.4%). There was only one outlier in the Bland–Altman plot (Figure 4D).

**FIGURE 5:**
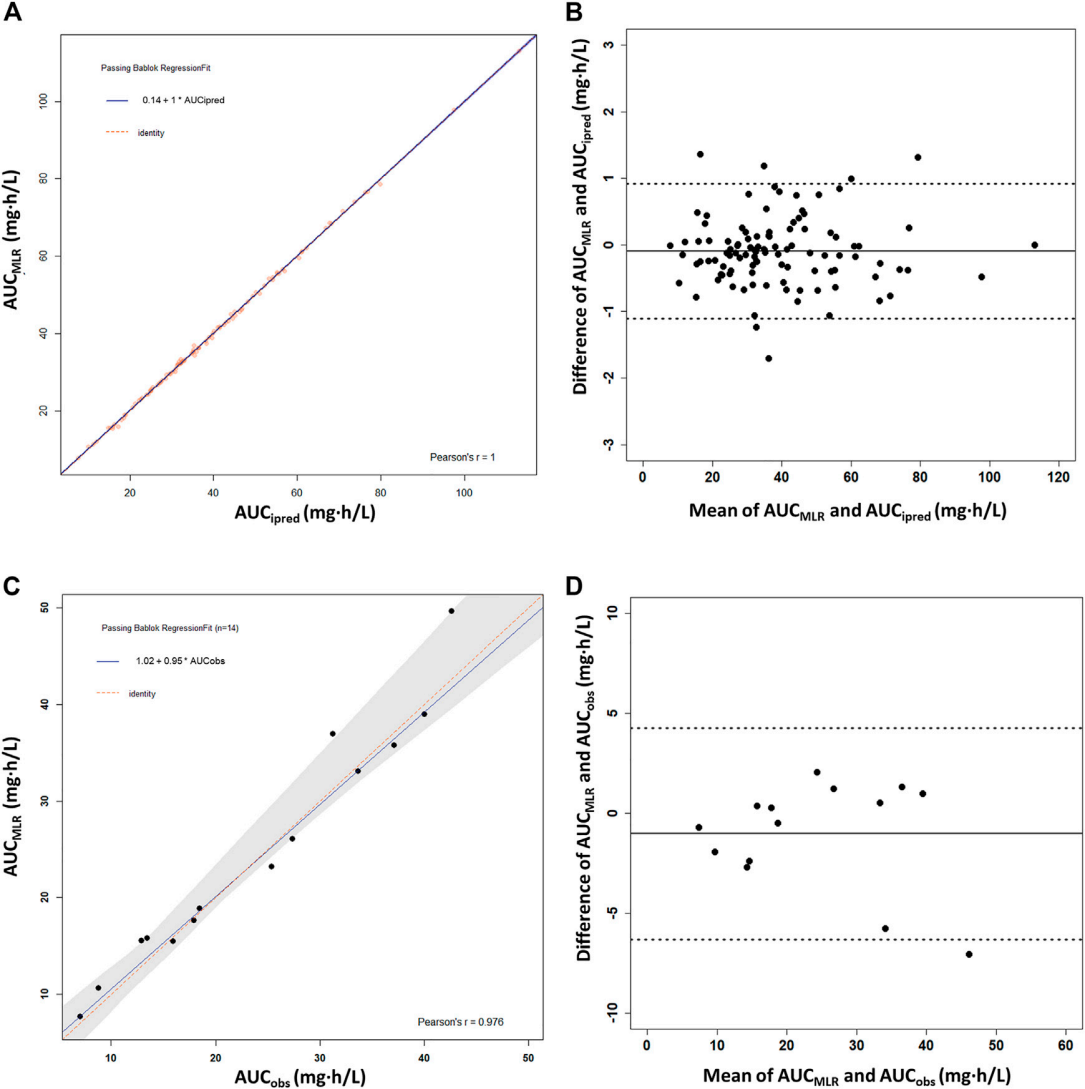
Passing–Bablok regression analysis and the Bland–Altman plot between the reference AUC_0–12h_ and the calculated AUC_0–12h_ of MPA obtained using the multiple linear regression model (AUC_MLR_) in the heart transplant recipients. **(A,B)** AUC_0–12h_ obtained by PPK prediction as the reference AUC_0–12h_ (AUC_ipred_) (*n* = 91). **(C,D)** Observed AUC_0–12h_ values from the patients with intensive data as the reference AUC_0–12h_ (AUC_obs_) (*n* = 14). AUC_0–12h_, the area under the curve of the dosing interval; MPA, mycophenolic acid.

The MLR analysis showed several one-to four-timepoint linear models with *R* ^2^ > 0.9 (Supplementary Table S3). Considering estimation reliability, collinearity, and convenience of clinical application, a best-fit linear equation with four timepoints was finally chosen: AUC_0–12h_ = 3.539 × C_0_ + 0.288 × C_0.5_ + 1.349 × C_1_ + 6.773 × C_4.5_ (*r* ^2^ = 0.999). No intercept was included in the linear equation because it was not significant (*p* = 0.471). High correlations between the PPK-predicted, linear-calculated, and observed AUC_0–12h_ values indicated the accuracy of the linear equation, with slopes of 1.0 (*r* = 1.00) and 0.95 (*r* = 0.976), respectively (Figures 5A,C). The %ME and %RMSE values between AUC_ipred_ and AUC_MLR_ were 0.33 and 1.75%, respectively. Compared with the AUC_obs_, the %ME and %RMSE values were 6.1 and 11.0%, respectively. The Bland–Altman plot showed that only six AUC_MLR_ values (5.7%) were outside the 1.96SD limits with AUC_ipred_ as the reference (Figure 5B). With AUC_obs_ as a reference, there was only one outlier calculated by the linear equation (Figure 5D). The linear sampling times also showed adequate accuracy of AUC_0–12h_ estimation by MAPB estimation (Supplementary Table S2).

## Discussion

To the best of our knowledge, this is the first study to report significant covariates affecting MPA pharmacokinetics in heart transplant recipients and provide a complete strategy for MPA exposure estimation using both the PPK modeling approach and linear regression. A two-compartment model with T_lag_ and three covariates adequately fitted the data. MPA exhibited EHC (Bergan et al., 2021). However, the EHC compartment was often supported by intensive data (Jiao et al., 2008; Wang et al., 2017; Colom et al., 2018), and it was validated to be inferior to the conventional two-compartment model (Zhang et al., 2019). Moreover, a two-compartment model with T_lag_ is more often chosen in organ transplant recipients (Kiang and Ensom, 2018; Yu et al., 2017). The two-compartment model without EHC in the current study adequately described the main part of the pharmacokinetic profile. However, there were still some deviations in the estimation of PK parameters. The large relative standard error (RSE%) and shrinkage of V_2_/F were probably caused by the inadequate fitness of the peak concentrations. Meanwhile, the parameters of the peripheral compartment (Q/F and V_3_/F) also had higher RSE% and shrinkage because the second peaks (or rebound concentrations) after 6 h post-dose, especially in the intensive data, were not well-fitted by the two-compartment model. In addition, the different sample collection times in the intensive and sparse data might have contributed to the high shrinkage in T_lag_. Nevertheless, the AUC _0–12h_ estimation accuracy was acceptable based on the evaluation results of the final PPK model.

Previously, no covariate had been found in heart transplant recipients with Bayesian and nonparametric analysis (Woillard et al., 2015). However, significant factors influencing the MPA pharmacokinetics are essential in explaining the large inter-individual variability and could guide dose initiation and adjustment. We found three significant covariates in the final PPK model, including PPI co-medication (on F), eGFR (on CL/F), and ALB (on V_2_/F). PPI co-medication and eGFR were powerful covariates, which resulted in significant changes in AUC_0–12h_ values, whereas the impact of ALB on AUC_0–12h_ was limited.

PPIs are routinely used as co-medications to prevent gastrointestinal tract complications following surgery. The drug–drug interaction (DDI) between MPA and PPIs was previously reported (Wedemeyer and Blume, 2014; Benjanuwattra et al., 2020). In heart transplant recipients with PPI co-medication, the AUC_0–12h_ of MPA was 25∼30% lower (Kofler et al., 2009a, 2009b). A similar reduction in the AUC_0–12h_ values was estimated in our final PPK model (27.6% decrease). A previous study found that parenteral PPI administration decreased MPA AUC_0–12h_ by 17.4% compared with oral administration in heart transplant recipients (Urbanowicz et al., 2020). However, we did not find a difference in F between different methods of PPI administration.

Moreover, diuretics also are commonly used medications after heart transplant surgery. Therefore, in our dataset, half of the patients received PPIs and diuretics during the study, three of whom were administered only diuretics without PPIs and five of whom received only PPIs without diuretics. Most patients took PPIs and diuretics simultaneously, especially during the period after surgery. The OFV (−18.4, *p* < 0.01) significantly decreased when diuretic co-medication on CL/F was included alone. However, it could not be retained in the full model once PPI co-medication on F was included. The impact of diuretics on the pharmacokinetics of MPA has to be investigated in further studies.

The published covariates with significant effects on CL/F of MPA in liver and renal transplant recipients include WT, Scr, SLCO1B1, and MRP-2 genetic polymorphism (Musuamba et al., 2012; Han et al., 2014; Yu et al., 2017). However, there was no significance in our study when including WT alone, while eGFR was a complex index to reflect renal function including WT, Scr, age, and sex. The effect of renal function on MPA CL/F was also found in previous studies, which showed that lower creatinine clearance is related to lower MPA clearance (Han et al., 2014; Langers et al., 2014). With severe renal function impairment, which possibly occurs after surgery, MPA exposure could increase by 30%. However, co-administration of PPIs decreased AUC_0–12h_ by approximately 30%, which resulted in adequate MPA exposure. For patients with mild renal impairment taking PPIs, AUC_0–12h_ only reached 45.7 mg h/L. In patients with normal renal function, AUC_0–12h_ reduced to 37.2 mg h/L, close to the lower limit of MPA exposure for rejection (Table 3). Attention should be paid in the case of PPI co-medication and renal impairment, especially for the initial dose and dose adjustment of MMF, in heart transplant recipients. When the renal function recovered to normal and PPI medication was stopped, the AUC_0–12h_ was still enough. If PPIs were still co-administered, the exposure of MPA would be lower than the target range. The recipient with ARC was at a higher risk of insufficient exposure to MPA. A dose adjustment might be necessary for this situation.

In previous studies, a series of covariates, including low plasma albumin levels and accumulation of the inactive MPAG, decreased the binding of MPA to ALB. WT and ALB were found to significantly influence MPA clearance and distribution volume or the protein-binding rate in renal transplant recipients (de Winter et al., 2010; Sheng et al., 2020). The subsequent increase in unbound MPA produces an increase in MPA clearance, resulting in decreased MPA exposure (de Winter et al., 2010). ALB was negatively related to CL/F of MPA in the liver transplant recipients, leading to lower MPA exposure (Langers et al., 2014). The effect of ALB on V_2_/F in our final model demonstrated the same trend of MPA concentrations. However, its impact seemed limited to the AUC_0–12h_. Other potential covariates were also investigated. The DDI between MPA and TAC was controversial in previous studies in healthy volunteers and renal transplant recipients (Kawauchi et al., 2019; Kim et al., 2018; Rong et al., 2019). However, in the current study, TAC trough concentrations and daily dose did not significantly influence the MPA pharmacokinetics. Other significant individual covariates, such as WT (on V_2_/F), PPIs (on Ka), and diuretics (on CL/F), were not included in the final PPK model, once other significant covariates were involved. The other potential covariates, such as age, POT, and liver function, were not found to significantly influence pharmacokinetic parameters. In addition, the Chinese patients in the present study received a similar MPA exposure at a lower dose of MMF (250–750 mg bid) compared with the Caucasian patients (750–1,500 mg bid) [35.2 (7.7–113.0) mg h/L vs. 33.8 (4.1–98.7) mg h/L] (Woillard et al., 2015). A 2- to 3-fold higher dosage resulted in similar MPA exposure in the Caucasian patients. A higher dose-normalized AUC_0–12_ was observed in the Chinese patients, which was also observed in a renal transplant population (Li et al., 2014). The lower WT in the Chinese population may be one of the reasons underlying the ethnic difference. However, other reasons, such as diet, pharmacogenomics, and enterohepatic circulation of MPA, may also contribute to this ethnic difference (Bergan et al., 2021).

In the ED-optimal sampling times, any two optimal sampling times could result in accurate AUC_0–12h_ estimation (Supplementary Table S2). However, if only one sample could be collected, the pre-dose sample was recommended. Sampling only at 4 h post-dose could have a high coefficient but larger bias (%ME of −7.5%). If only one sample at 1 h post-dose was collected, the coefficient could be as low as 0.7168. The optimal sampling times were validated in the observed intensive dataset. However, considerable precision (%RMSE of 32.4%) was observed because there were only 14 patients with intensive data. The PK profiles of these patients also showed increased between-subject variability.

The following linear regression model was generated based on the simulated data of the final PPK model: AUC_0–12h_ = 3.539 × C_0_ + 0.288 × C_0.5_ + 1.349 × C_1_ + 6.773 × C_4.5_. Although MLR models 2, 3, and 5 showed adequate accuracy of AUC_0–12h_ estimation with VIF <10, MLR model 5 required a shorter collection time (within 4.5 h post-dose) (Supplementary Table S3). A linear model using three timepoint concentrations of C_1_, C_2_, and C_4_ with an intercept of 23.56 for the prediction of AUC_0–12h_ in heart transplant recipients co-medicated with CsA or TAC was generated. However, an increased bias was found during clinical practice when calculating AUC_0–12h_ (Wada et al., 2007). Kaczmarek et al. (2008) established the more reliable multiple linear models of four timepoints (C_1_, C_4_, C_8_, and C_10_) without intercept (*r* ^2^ = 0.95); meanwhile, using the first 2-h post-dose concentrations [C_0.5_, C_1_ and C_2_ (*r* ^2^ = 0.84) or C_0.5_ and C_2_ (*r* ^2^ = 0.75)] could also provide an optional model to estimate AUC_0–12h_ (Kaczmarek et al., 2008). However, in our study, collecting samples at the absorption phase (C_0.5_ and C_1_) was not recommended for linear or MAPB estimation. The correlation efficient was only 0.7473 (Supplementary Table S2). Moreover, the MPA concentrations measured by the EMIT were higher than the results measured by HPLC (Kunicki et al., 2015; Bergan et al., 2021). Furthermore, we compared our linear equation with another linear equation based on Chinese adult heart transplant recipients. The MPA concentrations were determined by HPLC, and the four-timepoint equation (C_0.5_, C_2_, C_4_, and C_6_) had an intercept of 8.424 (Xiang et al., 2021). Interestingly, taking our simulation data as a reference, the calculated AUC_0–12h_ values by the published linear equation were generally higher by approximating the intercept (8.424), demonstrating that the bioanalysis methods’ systematic error probably caused the intercept. It still needs further validation.

The sampling times of the linear equation were around the ED-optimal sampling times. The ED-optimal sampling strategy and linear regression found similar time-point patterns. Four timepoints in the MLR equation could estimate AUC_0–12h_ accurately (*r* = 0.9699). However, collecting at 0.5, 1, and 4.5 h post-dose without the pre-dose sample could also obtain an accurate AUC_0–12h_ estimate (*r* = 0.9981). It meant that once the pre-dose was missing, AUC_0–12h_ estimation could still be obtained based on other samples by MAPB estimation. One sample alone (except for pre-dose) was not recommended for MAPB estimation. There are some recommendations for the use of the linear model and the final PPK model. The final PPK model and the linear equation were both accurate to estimate MPA AUC_0–12h_. However, the linear model’s premise was the exact collection timepoint and the EMIT bioanalysis method. Once there are missing samples, changes in co-medication or renal impairments require dose adjustment, and the PPK model is preferred for its flexible application and strong adaptability. Once the concentrations were obtained, prediction could be performed directly using the final PPK model, such as the online Bayesian estimator (Woillard et al., 2015). However, when covariates change (e.g., PPI co-administration ceases), the PPK simulation could give some guidance for dose adjustment.

This study had some limitations. Due to the limited number of participants, the patient groups could not represent all patients. Therefore, probably not all significant covariates could be identified. The intensive data were collected in only 14 patients, and hence our conclusions need to be externally validated with further intensive data. The linear model was based on the EMIT data, so MPA concentrations measured by HPLC cannot be used in our linear models. Finally, the applied immunosuppressant was TAC, with no patients taking cyclosporine, which could affect MPA exposure.

## Conclusion

A PPK model of MPA was established with three covariates. Co-medication of PPIs and eGFR had significant impact on MPA AUC_0–12h_. The optimal sampling times for MAPB estimation were pre-dose and 1 and 4 h post-dose. The following MLR model was generated: AUC_0–12h_ = 3.539 × C_0_ + 0.288 × C_0.5_ + 1.349 × C_1_ + 6.773 × C_4.5_. These models accurately calculated AUC_0–12h_ values. The PPK model is preferred for its flexible application and strong adaptability, while the linear regression equation was a convenient alternative for the fast estimation of MPA AUC_0–12h_ values.

## Data Availability

The raw data supporting the conclusions of this article will be made available by the authors, without undue reservation.
