# Supplementary material for: Estimation of Mycophenolic Acid Exposure in Heart Transplant Recipients by Population Pharmacokinetic and Limited Sampling Strategies
Source: Front Pharmacol. 2021 Nov 19;12:748609. doi: 10.3389/fphar.2021.748609 (PMC8640522; doi:10.3389/fphar.2021.748609)
Supplement: Supplementary file 1 [file Table1.DOCX]

**Supplementary Materials**

TABLE S1. The model building process of mycophenolic acid (MPA) in adult heart transplant recipients.

| PK Model # | Description | OFV | Degrees  of freedom | △OFV  based on | △OFV | Significance^#^ |
| --- | --- | --- | --- | --- | --- | --- |
| Base Model |  |  |  |  |  |  |
| 1 | One on compartment | -4687.4 | - | - | - |  |
| 2 | Two on compartment (base model) | -4720.4 | - | - | - |  |
| 3 | Two on compartment with EHC compartment | R | - | - | - |  |
| Forward inclusion | |  |  |  |  |  |
| 4 | BW on CL | -4728.2 | 1 | Model 2 | -7.8 | * |
| 5 | Scr on CL | -4726.8 | 1 | Model 2 | -6.3 |  |
| 6 | eGFR on CL | -4732.5 | 1 | Model 2 | -12.1 | ** |
| 7 | FKCONC on CL | -4726.8 | 1 | Model 2 | -6.4 |  |
| 8 | TBIL on CL | -4726.1 | 1 | Model 2 | -5.7 |  |
| 9 | POT on CL | -4713.3 | 1 | Model 2 | 7.1 |  |
| 10 | PPI on F | -4736.4 | 1 | Model 2 | -16.0 | ** |
| 11 | PPI on Ka | -4753.1 | 1 | Model 2 | -32.7 | ** |
| 12 | WT on V_2_ | -4730.1 | 1 | Model 2 | -9.7 | * |
| 13 | HCT on V_2_ | -4731.8 | 1 | Model 2 | -11.3 | ** |
| 14 | POT on V_2_ | -4723.6 | 1 | Model 2 | -3.2 |  |
| 15 | WT on Q | -4729.4 | 1 | Model 2 | -9.0 | * |
| 16 | POT on Q | -4717.9 | 1 | Model 2 | 2.5 |  |
| 17 | POT on V_3_ | R | - | - | - |  |
| 18 | Diuretics on CL | -4738.8 | 1 | Model 2 | -18.4 | ** |
| 19 | Diuretics on CL, PPI on F | -4739.5 | 1 | Model 18 | 0.7 |  |
| 20 | ALB on V_2_ | -4753.6 | 1 | Model 2 | -33.2 | ** |
| 21 | ALB on V_2_, eGFR on CL | -4765.9 | 1 | Model 20 | -12.3 | ** |
| 22 | ALB on V_2_, eGFR on CL, PPI on F (Full model) | -4779.1 | 1 | Model 21 | -13.2 | ** |
| Backward exclusion | |  |  |  |  |  |
| 23 | eGFR on CL, PPI on F (Remove ALB on V_2_ from Model 22) | -4750.0 | 1 | Model 22 | +29.1 | ** |
| 24 | eGFR on CL (Remove PPI on F from Model 23) | -4732.5 | 1 | Model 23 | +17.5 | ** |
| Final model |  |  |  |  |  |  |
|  | ALB on V_2_, eGFR on CL, PPI on F1 (Model 22) | -4779.1 | 1 | Model 2 | -58.7 | ** |

Ka: absorption constant; CL: clearance of the central compartment; F: the bioavailability; V_2_: distribution volume of the central compartment; Q: clearance of peripheral compartment; V_3_: distribution volume of the peripheral compartment; EHC: enterohepatic recirculation; R: minimization terminated; eGFR, estimated glomerular filtration rate; FKCONC: trough concentration of tacrolimus; Scr, serum creatine concentration; ALT, alanine aminotransferase; ALB, albumin; TBIL, total bilirubin; HCT, hematocrit; POT, postoperative time.

Noncontinuity covariates (such as SEX, co-medication) were implemented using Equation (2).

Continuous covariates (such as WT, Age, ALT, eGFR and Scr) were implemented using Equation (3) and (4).

# Significance: * p<0.01, **p<0.001

TABLE S2. Predictive performance of MPA AUC_0-12h_ estimation based on the proposed optimal sampling schedules in adult heart transplant recipients.

| Sample schedule # | Sampling times  (hours postdose) | No. of sampling times | Correlation coefficient (r^2^) | %ME | %RMSE |
| --- | --- | --- | --- | --- | --- |
| 1 | Predose, 1, 4 | 3 | 0.9981 | -0.1 | 2.9 |
| 2 | Predose, 1 | 2 | 0.9865 | 1.1 | 8.6 |
| 3 | Predose, 4 | 2 | 0.9846 | -2.7 | 10.5 |
| 4 | 1, 4 | 2 | 0.9918 | -1.5 | 6.9 |
| 5 | Predose | 1 | 0.9768 | -0.2 | 11.8 |
| 6 | 1 | 1 | 0.7168 | -7.4 | 38.1 |
| 7 | 4 | 1 | 0.9588 | -7.5 | 19.3 |
| 8 | Predose, 0.5, 1, 4.5 | 4 | 0.9699 | -1.0 | 9.3 |
| 9 | Predose, 0.5, 1 | 3 | 0.9885 | 2.0 | 8.6 |
| 10 | Predose, 0.5 | 2 | 0.9817 | -0.5 | 10.5 |
| 11 | Predose, 4.5 | 2 | 0.9833 | -2.8 | 10.8 |
| 12 | 0.5, 1, 4.5 | 3 | 0.9981 | -1.3 | 3.7 |
| 13 | 0.5, 1 | 2 | 0.7473 | -5.3 | 36.2 |
| 14 | 0.5 | 1 | 0.6875 | -9.7 | 40.4 |
| 15 | 4.5 | 1 | 0.9634 | -7.5 | 18.4 |

TABLE S3. The multiple linear regression models based on the simulated MPA concentrations in adult heart transplant recipients

| MLR Model # | R^2^ | Independent variables | Coefficient (Beta) | SE | *P* value | Variance inflation factor (VIF) |
| --- | --- | --- | --- | --- | --- | --- |
| 1 | 0.899 | Intercept | 8.534 | 1.146 | 0.000 |  |
|  |  | C_6_ | 11.773 | 0.387 | 0.000 | 1.00 |
| 2 | 0.996 | Intercept | 0.623 | 0.293 | 0.036 |  |
|  |  | C_6_ | 9.839 | 0.091 | 0.000 | 1.25 |
|  |  | C_1_ | 1.804 | 0.038 | 0.000 | 1.25 |
| 3 | 0.997 | Intercept | 1.113 | 0.256 | 0.000 |  |
|  |  | C_6_ | 8.697 | 0.187 | 0.000 | 7.58 |
|  |  | C_1_ | 1.707 | 0.035 | 0.000 | 1.51 |
|  |  | C_0_ | 1.453 | 0.218 | 0.000 | 8.74 |
| 4 | 0.998 | Intercept | 0.586 | 0.239 | 0.016 |  |
|  |  | C_6_ | 4.201 | 0.774 | 0.000 | 172.64 |
|  |  | C_1_ | 1.676 | 0.031 | 0.000 | 1.56 |
|  |  | C_0_ | 2.625 | 0.273 | 0.000 | 18.34 |
|  |  | C_4.5_ | 3.349 | 0.564 | 0.000 | 103.52 |
| 5 | 0.999 | Intercept | -0.096 | 0.133 | 0.471 |  |
|  |  | C_1_ | 1.349 | 0.024 | 0.000 | 2.94 |
|  |  | C_0_ | 3.539 | 0.087 | 0.000 | 5.69 |
|  |  | C_4.5_ | 6.773 | 0.072 | 0.000 | 5.17 |
|  |  | C_0.5_ | 0.288 | 0.017 | 0.000 | 2.52 |
