# Supplementary figures and images for: Estimation of Mycophenolic Acid Exposure in Heart Transplant Recipients by Population Pharmacokinetic and Limited Sampling Strategies
Source: Front Pharmacol. 2021 Nov 19;12:748609. doi: 10.3389/fphar.2021.748609 (PMC8640522; doi:10.3389/fphar.2021.748609)

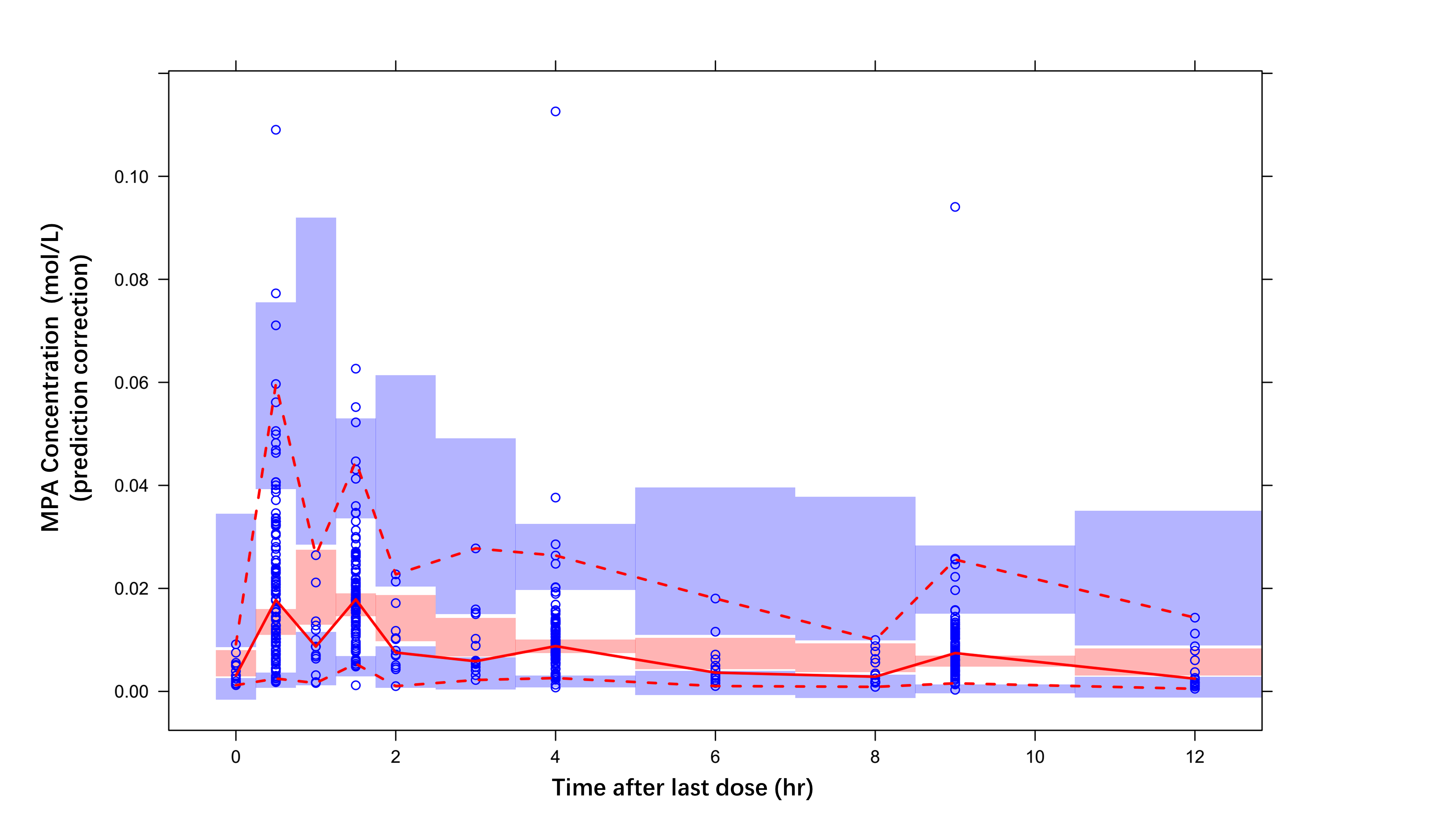

Supplement: Supplementary file 2 [file Image1.TIF]

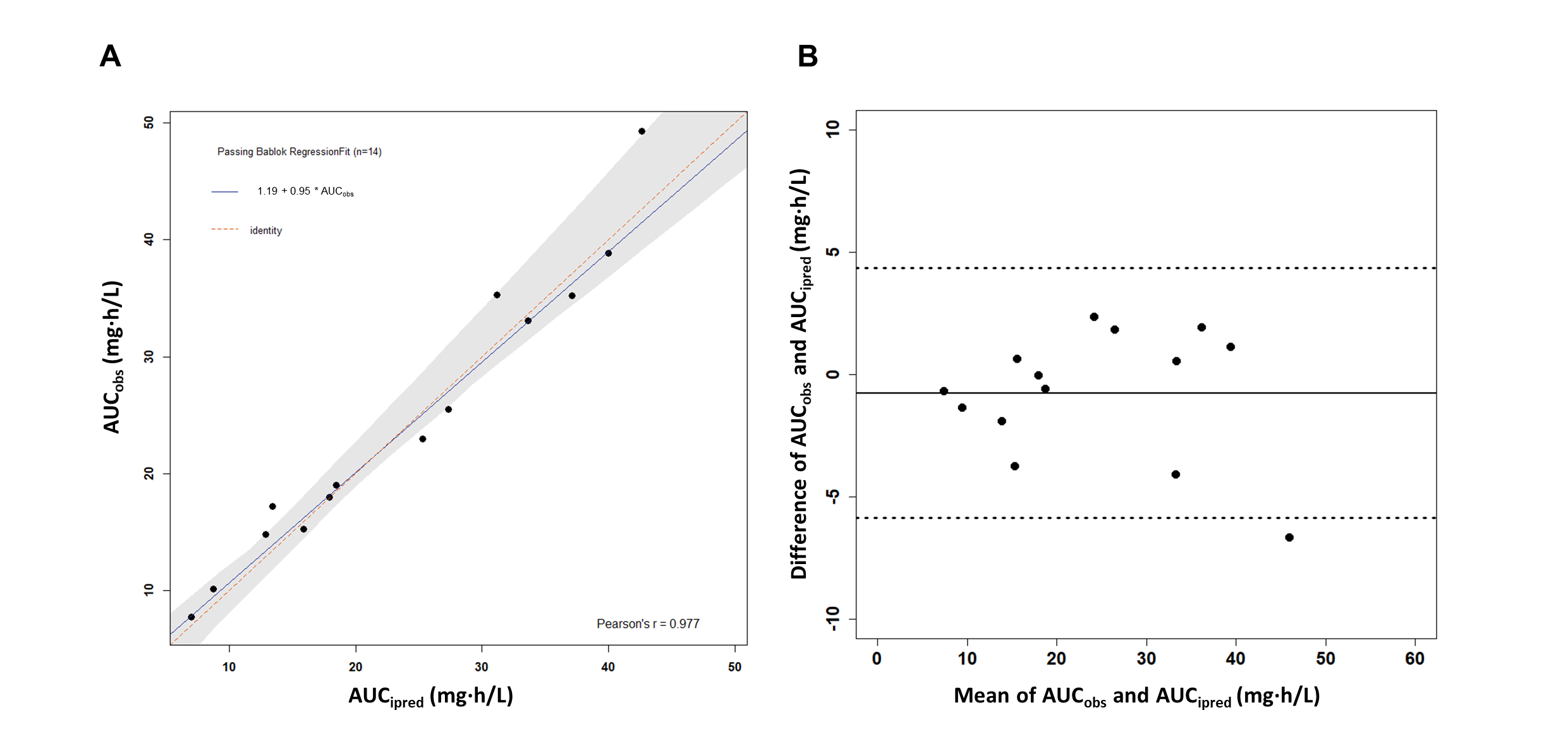

Supplement: Supplementary file 3 [file Image2.TIFF]
